# Synthesis of High Fluorescent Carbon Dots by Laser Ablation of Bay Leaves in Biocompatible Solutions

**DOI:** 10.1002/bio.70202

**Published:** 2025-05-23

**Authors:** L. Torrisi, M. Cutroneo, A. Torrisi, D. Manno, A. Serra

**Affiliations:** ^1^ MIFT Department, Physics University of Messina Messina Italy; ^2^ Medicine and Surgery Department Kore University of Enna Enna Italy; ^3^ Department of Mathematics and Physics E. De Giorgi University of Salento Lecce Italy

**Keywords:** bay leaves, biodispersion, carbon dots, laser ablation, luminescence, optical spectroscopy

## Abstract

A green dispersion of carbon dots (CDs) can be obtained by laser irradiation of bay leaves in liquids. The CDs synthesis is obtained using an Nd:YAG laser irradiating multilayered leaves placed into a phosphate‐buffered saline (PBS) solution. The nanoparticle generation and their functionalization by the solution salts produce a high‐intensity dispersion luminescence in the visible region, which is induced by UV excitation. The ablation uses ns pulses with 100 mJ energy employed with a 1 Hz repetition rate focused on the vegetal target. Plasma investigations, UV–visible, and IR optical spectroscopies were employed. Morphological, optical properties, and other characteristics of the dispersion are presented and discussed. The synthesized CDs showed an absorption peak at 274 nm to confirm the p–p* transition of the carbon core state, while the CD particles were spherical with a size of less than 10 nm. The CDs fluorescent emission is in the blue region, around 472 nm, upon excitation at 365 nm. The synthesized CDs showed stability over a long period (3 months). This study provides an inexpensive, green, and simple method for CDs synthesis for important biocompatible dispersion applications in different scientific fields, especially from biology to medicine.

## Graphical abstract

The pulsed laser ablation of bay leaves in phosphate‐buffered saline (PBS) solution synthesizes carbon dots (CDs) with an ablation yield of about 0.44 μg carbon/pulse. The CDs are luminescent under UV irradiation, emitting mainly at 472 nm, with a luminescence yield of about 90%. CD dispersion can be employed efficiently in biology and medicine for its high biocompatibility and stability.

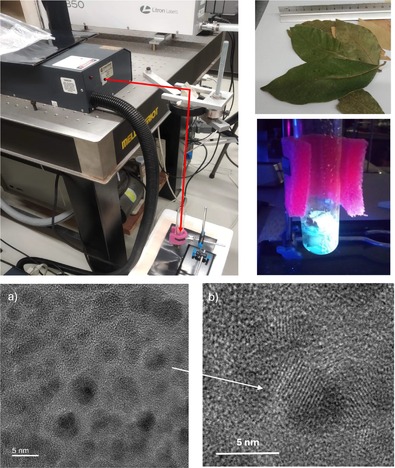

## 1 Introduction

Luminescent materials are more and more investigated for their old and innovative applications in optoelectronic, sensing, detectors, and bio‐imaging devices due to their high fluorescent quantum yield (QY) [1]. Different fluorescent materials use semiconductor quantum dots (QDs), compounds of gold, silver, lead, silicon, and others [2].

In the last few years, innovative QDs based on carbon have been obtained with the technique of bottom‐up using an electrochemical synthesis approach or a top‐down technique using laser ablation in liquids [3]. Overall, the interest is devoted to luminescent biocompatible solutions useful for biological and medical applications. Bioimaging in cell cultures, drug transport, internalization of nanometric nanoparticles in cells, thermal therapy, and radiotherapy find significant advancements using such low‐dimensional nanoparticle dispersions [4].

In particular, a strong interest is devoted to the use of biocompatible and nontoxic luminescent carbon dots (CDs), a class of very promising material with properties similar to those of semiconductor QDs [5].

CDs can be synthesized not only using carbon targets such as graphite and graphenes but also using green targets such as vegetable charcoal, plants, and leaves of different types, citrus and fruit, coming from different geographic areas [5, 6].

When having a size lower than 10 nm, the carbon nanoparticles are recognized as “carbon quantum dots,” having peculiar luminescence properties due to their high surface‐to‐volume ratio. Generally, CDs contain a high amount of diamond‐type sp3 hybridization of carbon atoms along with graphite‐type sp2 hybridization. They can contain elementary constituents such as graphene, graphene oxide, amorphous carbon, onion‐like carbon, nanotubes, diamond, and carbon structures in which different functional oxygen groups and impurities are present [7].

In contrast to the electrochemical bottom‐up approach, the top‐down processes are based on the nucleation of individual atoms in a laser‐generated plasma. The CDs synthesis has been performed using egg white, honey, sweet potato, bamboo leaves, orange and lemon juice, banana juice, tea extract, and others [8, 9].

This study puts in evidence the high luminescence obtained by synthesizing CDs from bay leaves using the top‐down approach based on laser ablation in a widely used biological solution, the phosphate‐buffered saline (PBS) solution, as reported in the literature [10, 11].

The benefits of bay leaves have been known since ancient times. They are nutritional and antioxidant and contain vitamin A, vitamin B6, and vitamin C, known to support a healthy immune system and as a digestive aid [12]. The tea is also very aromatic, which can help relieve sinus pressure or a stuffy nose. The bay leaves contain many carbohydrates and valuable substances such as eugenol, limonene, lauric acid, and flavonoids and contain about 1.3% essential oils, comprising 45% eucalyptol, 8%–12% terpinyl acetate, 12% other terpenes, 3%–4% sesquiterpenes, 3% sesquiterpenes, 3% methyl eugenol, and other linalool, phellandrene, geraniol, terpineol, and lauric acid [13]. Literature reports that the bay leaves' chemical content consists of a moisture content of 4.95%, protein content of 7.62%, crude oil of 8.57%, ash content of 3.63%, crude fiber content of 24.40%, and total carbohydrates of about 50.83%. The mineral element contents are Ca (377 mg/100 g), P (112 mg/100 g), K (550 mg/100 g), Fe (45 mg/100 g), Cu (0.63 mg/100 g), Mg (112 mg/100 g), Mn (7.313 mg/100 g), and Zn (2.90 mg/100 g) [12].

The high energy of the laser pulse, incident on the bay leaves placed in the PBS solution, generates a plasma at the solid–liquid interface, which expands in the liquid and contains atoms and molecules ablated at high speed and kinetic energy. Before the bubble implodes, atoms, ions, and molecules collide energetically, producing low‐dimensional nanostructures and nanoparticles consisting mainly of carbon [14]. The result is a generation of CDs dispersion, which results in very biocompatible, nontoxic, simple, cheaper, and free from any type of contaminants, ready to be injected into cells, tissues, and organs for bioimaging and other utilities. The presented results are preliminary to a further deeper investigation, which will be performed to investigate the CDs size, shape, surface functionalization, and atomic structure, as reported in similar literature data [15].

## 2 Material and Methods

An Nd:YAG laser operating in fundamental frequency at 1064 nm, 3 ns pulse duration, 100 mJ pulse energy, 1 mm^2^ spot size, and 1 Hz repetition rate was employed for laser ablation. The laser fluence corresponds to 10 J/cm^2^, while the intensity corresponds to 3.3 × 10^8^ W/cm^2^. The laser beam is generated horizontally on an optic table at 1 cm^2^ spot size, arriving in a prism, is deflected vertically towards the bottom, is focused by a 50 cm focal lens at 1 mm^2^ spot size, and is incident on the target placed in a glass test tube inside which it is immersed in liquid. Irradiation times of 2 h at 1 Hz have been performed using 7200 laser pulses. The laser irradiation was performed in air at room temperature (20°C) and 1 atm pressure.

The target is constituted by two large bay leaves cut at about 1 cm^2^ surface and placed in a stack inside the glass test tube. The laurel plant used for its leaves lives near our city of Messina (Sicily, Italy). Using green and ripe leaves, their average thickness, evaluated by 20 measurements in different leaves, was 350 μm, while the density of about 0.25 g/cm^3^ agrees with the literature data [16, 17].

The stack of cut overlapping leaves consists of 20 layers, each approximately 350 μm thick. To avoid completely piercing the leaf pack, the target is moved during the laser ablation process.

**FIGURE 1:**
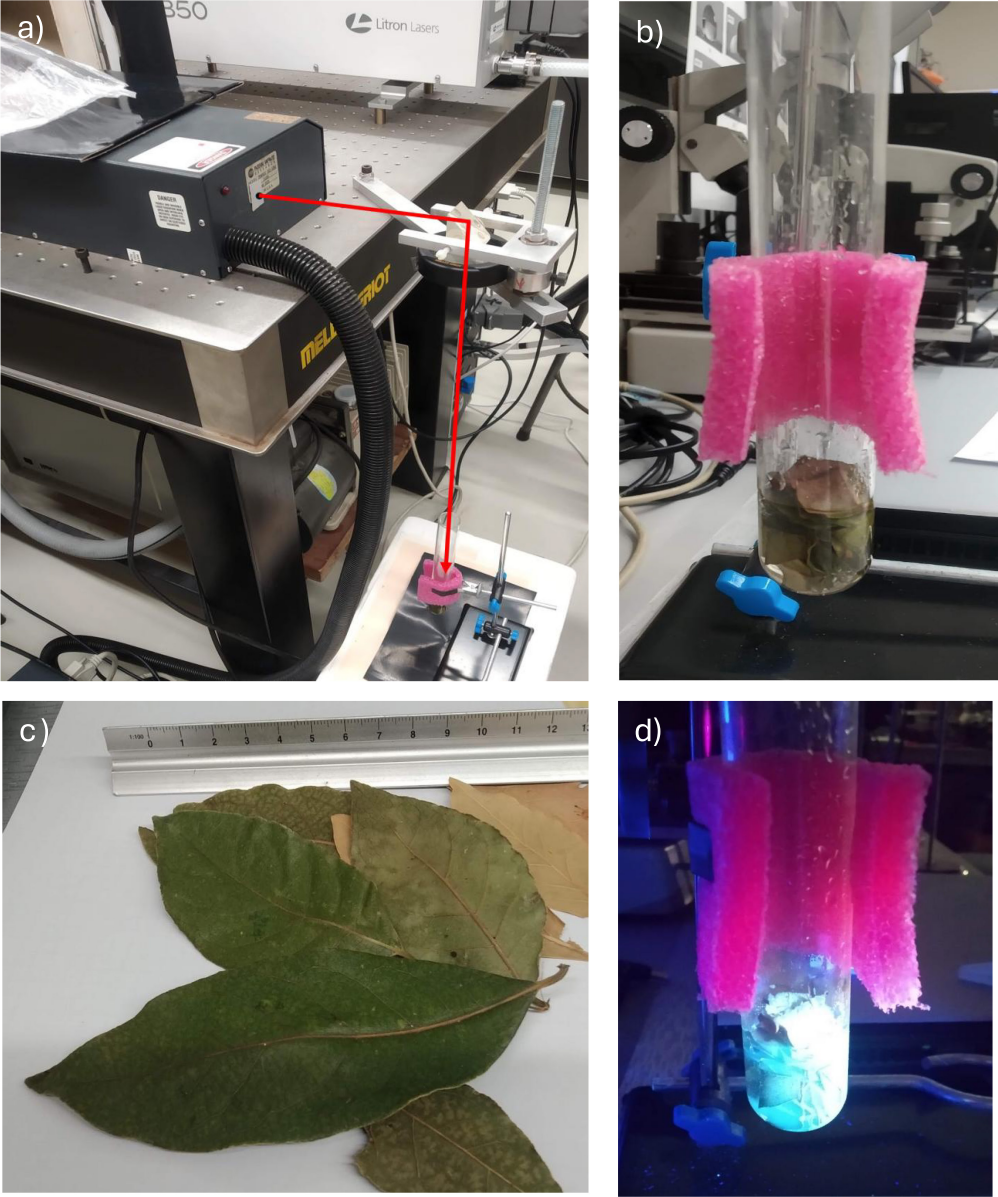
Experimental setup (a), target with PBS during irradiation (b), used bay leaves (c), and visible luminescence under UV excitation after the laser ablation process (d).

Figure 1 shows a photo of the experimental setup. The figure shows the used laser (Tempest), the target positioning into the glass tube, and the optical prism deflector with the focalizing lens (a), a zoom of the staked leaves inside the glass tube immersed in PBS solution (b), a photo of the used bay leaves (c) and a photo of the visible luminescence emitted in the blue region by the target after the laser irradiation (2 h at 1 Hz) under UV excitation.

The target was immersed in an 8 mL PBS solution. This is a tenfold concentrated blend of phosphate buffer and saline solution with a pH of 7.4 and RNase‐free, typically used in molecular biology [18]. The PBS solution was prepared by dissolving a 2 g PBS tablet, purchased from Sigma‐Aldrich [19], in a volume of distilled water of 200 mL. The PBS solution is not luminescent under UV irradiation.

The lamp used for UV excitation was the Blak‐Ray lamp (Model UVL‐56), emitting at 365 nm with a wavelength (full width at half maximum [FWHM]) of about ± 10 nm. The UV lamp source fluence was about 100 mJ/cm^2^.

The CDs dispersion of luminescence was investigated in wavelength emission (250–800 nm) using the optical spectrometer Avantes AvaSpec‐2048‐USB2 coupled to an optical fiber input. It features a 2048‐pixel CCD detector array, has 16 different gratings with varying dispersion and blaze angles, and is controlled by a simple portable PC.

The UV–Vis spectrometer (Model Jasco double beam V‐750) was used to perform transmittance and absorbance measurements in the 250–700 nm wavelength range with 0.1 nm resolution. This instrument employs quartz cuvettes as liquid containers and a precision double‐beam optical system. Simultaneously, it measures the sample and the reference beams, allowing for accurate compensation of background noise and drift.

The attenuated total reflectance coupled to the Fourier transform infrared spectrometer (ATR‐FTIR) (Model JASCO 4600) was employed in the 400–4000 cm^−1^ wavenumber region for IR spectroscopy with a 0.7 cm^−1^ resolution. ATR‐FTIR analyzed a depth of about 0.5–2.0 μm, depending on the sample composition, and gives detailed information on the transmittance and absorbance of the molecular groups present in the sample.

Optical microscopy was employed to obtain information on the morphology of the used target before and after the laser irradiation in PBS conditions.

TEM microscopy was employed to observe, at high magnification and resolution, the CDs morphology by a Jeol JEM‐ARM 200F NEOARM microscope at 200 kV. Samples for TEM observations were prepared by drop‐casting freshly prepared solutions containing CDs onto 400 mesh copper grids coated with Lacey Formvar/Carbon layers (TED PELLA Inc. USA) and dried slowly in natural air. The images were processed with Image Pro‐Plus software.

## 3 Results and Discussion

The laser ablation of the leaves in the PBS solution produces a leaf perforation successively to about 100 laser shots, indicating that the removed depth per laser shot is about 3.5 μm/pulse. The average hole has a 1.2 mm diameter; thus, the removed volume is about 4 × 10^−3^ mm^3^/pulse. Assuming a mass density of 0.25 g/cm^3^, it means that the leaf ablation yield Y evaluation is about:

(1) $$Y\left(\mathrm{bay}\;\text{leaf}\right)\approx 1\;\mathrm{\mu g}/\text{pulse}$$

Literature reports that the mean content of carbon in bay leaf is about 440 mg/g [20]. This assumption means that the carbon ablation yield is about:

(2) $$Y\left(\text{carbon}\right)\approx 0.44\;\mathrm{\mu g}/\text{pulse}$$

The investigation of the CDs dispersion luminescence was performed by analyzing the dispersion placed in a quartz container through a 300‐μm‐diameter optical quartz fiber. The liquid sample was illuminated with a white lamp and successively with the UV lamp at 365 nm, recording its luminescence with the Avantes spectroscopy.

**FIGURE 2:**
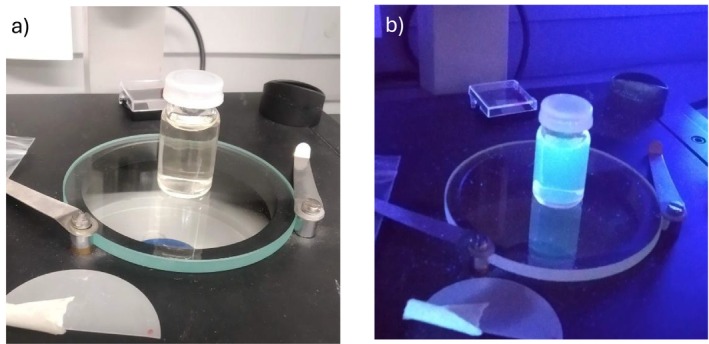
CDs dispersion observed in white light (a) and under UV light (b).

Figure 2 shows a photo of the prepared sample illuminated in a white laboratory lamp (a) and with a UV lamp (b), observing blue luminescence only in the second case.

On the basis of the evaluated ablation yield, the final CDs dispersion concentration C obtained after 2 h laser ablation (7200 laser pulses) is about:

(3) C=3.16mg/8mL=0.39mg/mL

**FIGURE 3:**
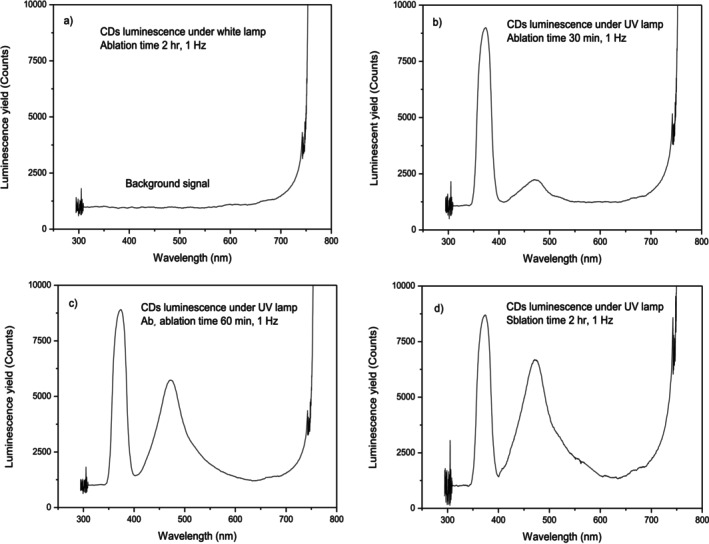
Optical luminescence yield versus wavelength in the non‐UV illuminated sample (a) and in that illuminated and produced after 30 min (b), 1 h (c), and 2 h (d) laser ablation time.

The CDs dispersion illuminated in the laboratory using white light and observing its emitted light does not show any luminescence, as observable from the background spectrum reported in Figure 3a. Instead, an illuminated under 365 nm UV lamp shows a luminescence proportional to the irradiation time, i.e., to the CDs dispersion concentration, as reported in the spectra of Figure 3b–d, obtained after a laser irradiation time of 30 min, 1 h, and 2 h, respectively.

Spectra show the UV peak centered at 365 nm with an FWHM of about 20 nm. This peak is intense depending on the distance of the dispersion from the lamp. It was revealed through the transmission of the liquid in the cuvette that, slightly decreases with increasing concentration of the dispersion due to the greater absorption in it. The luminescence peak is intense and large. It is centered at about 472 nm (maximum peak) with an FWHM of ± 47 nm. The peak is not perfectly Gaussian because it shows less slope towards longer wavelengths. Its yield, considered as luminescence subtended peak area, grows with the laser irradiation time, i.e., with the CDs concentration in the 8 mL BPS solution. The peak corresponds to the main energy of the photon emission of 2.63 eV, which represents the average band gap energy of the electronic structure excited by UV photons at 3.41 eV.

The luminescence emission, in fact, can be explained as consequent electron excitation from the valence to the conduction band, corresponding to π → π* transitions, and vice versa, deexcitation. The levels of excitation and deexcitation are enriched by the CDs surface states, which give rise to intrinsic absorption of *n* → π* transition and successively to vice‐versa transitions with luminescence emission [21].

The fluorescence QY, defined as the ratio of the number of photons emitted to the number of photons absorbed, can be better evaluated from the spectrum reported in Figure 3b, which reports a minor luminescence for the lower CDs concentration at 30 min irradiation time and a less absorption and scattering of the incident UV photons. In this case, a good QY preliminary evaluation can be given by comparing the area of the UV peak, centered at 365 nm, to that of the luminescence band centered at 480 nm, and a width of about ± 32 nm, and assuming an isotrope distribution of the absorbed and emitted photons. From such preliminary evaluation, the calculable percentage of the QY becomes:

(4) QY=photons emitted/photons absorbed≈25%

Increasing the laser irradiation time, the luminescence increases, but the UV peak becomes more absorbed and scattered by the nanoparticles in the liquid, decreasing in intensity.

**FIGURE 4:**
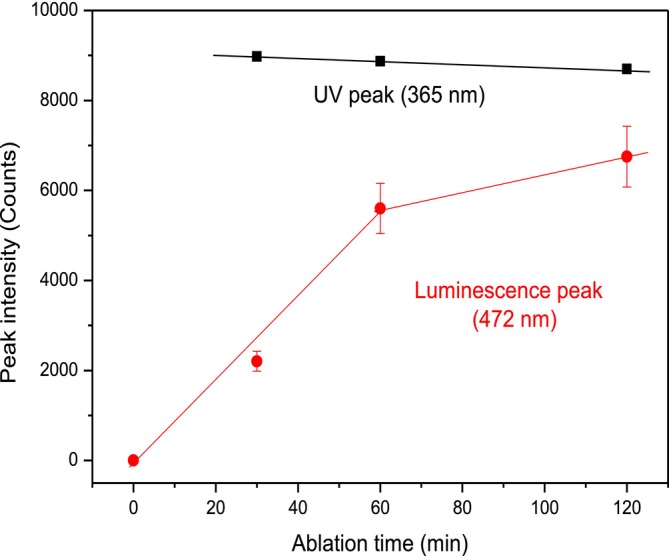
Luminescence peak intensity versus laser ablation time.

The luminescence plot versus the laser irradiation time is reported in Figure 4. It shows the linear enhancement of the CDs luminescence (measured error of about 10%) in the first hour of laser irradiation time and a saturation trend in the second hour of irradiation, while the exciting UV peak shows a small decrement with time. Results indicate that the luminescence grows nearly linearly with the CDs concentration, tending to saturate at high laser ablation times, while the exciting light exhibits a slight reduction due to the absorption into the solution with a higher CDs concentration.

Surprisingly, the reported photoluminescence (PL) spectra show that the CDs luminescence peak is comparable with the exciting UV peak, indicating a very high QY, understood as the ratio between the number of emitted photons per incident exciting photon [22]. Assuming the transmittance of the UV peak at 365 nm through the quartz cuvette to be of the order of 98%, while that of the luminescence peak centered at 472 nm to be about 100%, and assuming that a part of incident UV photons generate the background of the luminescence peak (~20%), at 2 h laser ablation the ratio between the two peaks gives, as a first approximation, a high QY of the order of 90%.

**FIGURE 5:**
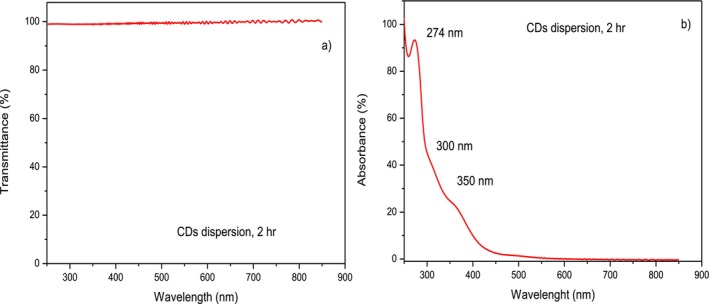
Transmittance (a) and absorbance (b) of the CDs dispersion prepared at 2 h laser ablation.

UV–visible spectroscopy has been employed to measure the transmittance and absorbance of the CDs dispersion in the cuvette using a double‐beam analysis. Measurements were performed from the near UV region, around 250 nm, to the visible region up to about 900 nm in the NIR region. The transmittance and absorbance spectra are reported in Figure 5a,b, respectively. The transmittance is nearly 100% in all the visible ranges, while the absorbance is negligible in the visible range but appears enhanced in the UV region by up to about 90% at 250 nm. Particularly interesting are the absorbance peak occurring at 274 nm and the two bending curves in the decay trend occurring at about 300 and 350 nm, indicating that small nanostructures are present in the analyzed liquid.

**FIGURE 6:**
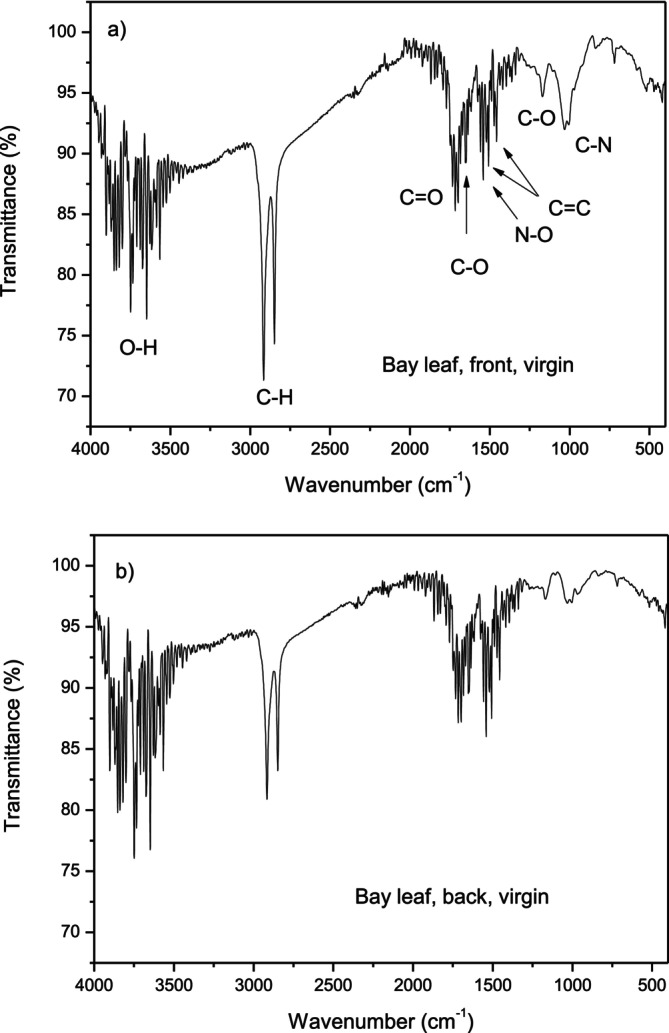
FTIR spectra of the bay leaf virgin in front (a) and in back (b) face.

The target of bay leaves was submitted, before the laser irradiation, to ATR‐FTIR spectroscopy to have information on the molecular groups present in its surface and matrix, which are responsible for the laser absorption and consequent ablation. The analysis has been performed on the front face, the greener colored, and the back face, the less green colored, of the leaf. Figure 6 reports the ATR‐FTIR spectrum relative to the frontal face (a) and the back face (b) of the bay leaf.

The bands between 3900 and 3500 cm^−1^ and at 1040 cm^−1^ can be attributable to O‐H stretching vibrations, indicating high hydroxyl groups in the structure of the leaf. The intense bands observed at around 2920 and 2845 cm^−1^ come from the stretching vibration of C‐H groups. Spectra also show the stretching vibrations of the C=O group from ketones observed at 1720 cm^−1^, the band at 1620 cm^−1^ corresponding to the stretching of the C‐O bond of amide‐I, and the weak band at 1440 and 1500 cm^−1^ corresponding to –C=C‐ vibrations from aromatic skeletal compounds. The bands at 1540 are due to N‐O groups. The bands at 1167 and 1033 cm^−1^ correspond to C‐O and C‐N groups, respectively. The peak identifications of the FTIR spectra agree with the literature [23, 24].

Substantially, the two faces have the same absorbent groups; however, the back face has minor C‐H, C‐O, and C‐N groups, as evident from the spectra comparison.

**FIGURE 7:**
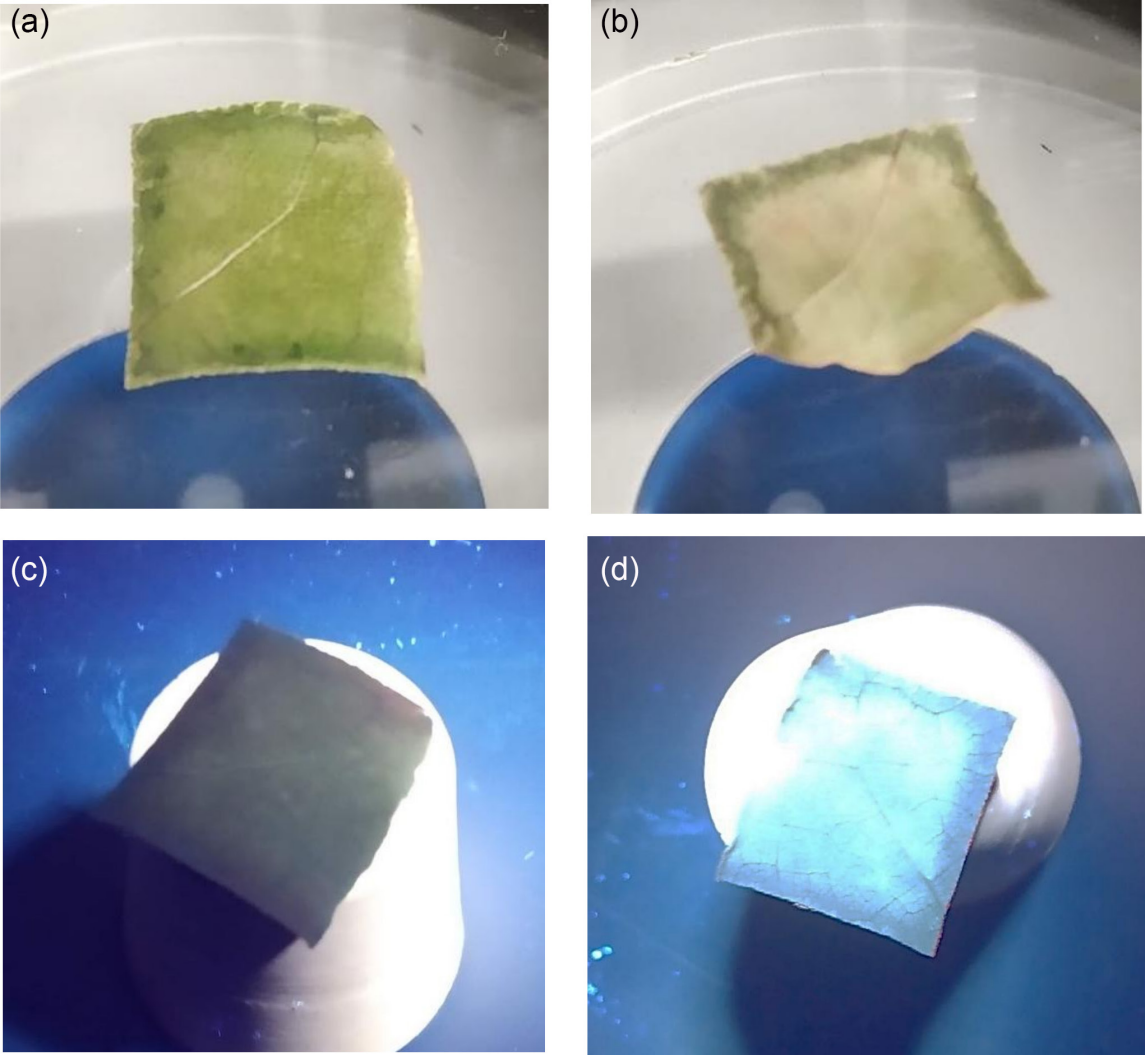
Microscope image of the bay leaf with CDs deposition on front face (a) and back face (b), of the virgin leaf without CDs under UV lamp irradiation (c), and of the leaf with CDs deposition emitting blue luminescence under UV lamp irradiation (d).

The laser ablation of the leaves in PBS has generated CDs, which are dispersed both in the liquid and also on the surfaces of the leaves, which appear luminescent under UV light excitation. Figure 7 shows a photo of the front (a) and back (b) faces of one leaf immersed in PBS, on which many CDs are deposited on its surface. Figure 7c reports a photo of the virgin foil, not immersed in PBS, under a UV lamp, which is not luminescent because without CDs, showing a dark image of its surface. Figure 7d shows a photo of the foil extracted from the solution after 2 h laser ablation on which CDs have been deposited on its surface and which, under UV lamp illumination, appears strongly luminescent in the blue region.

**FIGURE 8:**
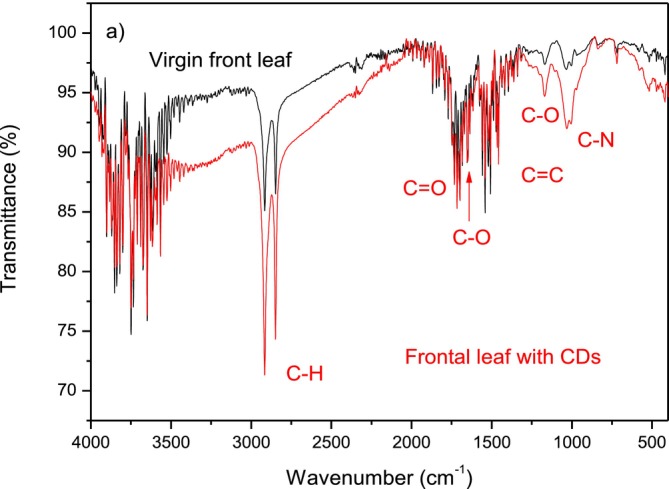
ATR‐FTIR spectra comparison between the virgin front leaf without CDs and the leaf with deposited CDs on its surface.

ATR‐FTIR analyses were performed on the bay leaves immersed in the PBS during the laser ablation, which produced nanoparticles that generated a CDs deposition on their surface. Figure 8 reports the FTIR spectra comparison between the leaves without and with the surface‐deposited CDs, observing the front side of the leaf.

The comparison shows that, in general, in the presence of CDs, the transmittance in the wavenumber region 4000–400 cm^−1^ decreases. The maximum decrement is in the region at higher wavenumbers; for example, at 3250 cm^−1^, the decrement is about 5.8% of the transmittance in the virgin leaf without CDs. The transmittance reduction is lower for minor wavenumbers; for example, at 1400 cm^−1^, it is about 1%.

The most surprising thing about the comparison is that the presence of CDs intensifies the transmittance peaks, i.e., the absorbance, found in the virgin leaf. In fact, the intense bands observed at around 2920 and 2845 cm^−1^ due to the vibration of the C‐H groups are intensified by about a factor of 200%. The stretching vibrations of the C=O group observed at 1720 cm^−1^ are intensified by about a factor of 3.7%. The band at 1620 cm^−1^ corresponding to the stretching of the C‐O bond is intensified by about 3%. The bands at 1440 and 1500 cm^−1^ corresponding to –C=C‐ vibrations are intensified by about a factor of 2%. The bands at 1167 and 1033 cm^−1^ due to the C‐O and C‐N groups are intensified by about a factor of 270% and 240%, respectively. Instead, the OH groups around 3900 and 3500 cm^−1^ remain nearly constant.

This comparison indicates the consistency of the high concentration of CDs after 2 h leaves laser ablation in PBS, permitting them to have information on the CDs molecular composition. A tentative analysis of this was performed by the subtraction of the previous two spectra, which permits having more detailed information on the nature of the synthesized CDs in the PBS solution.

**FIGURE 9:**
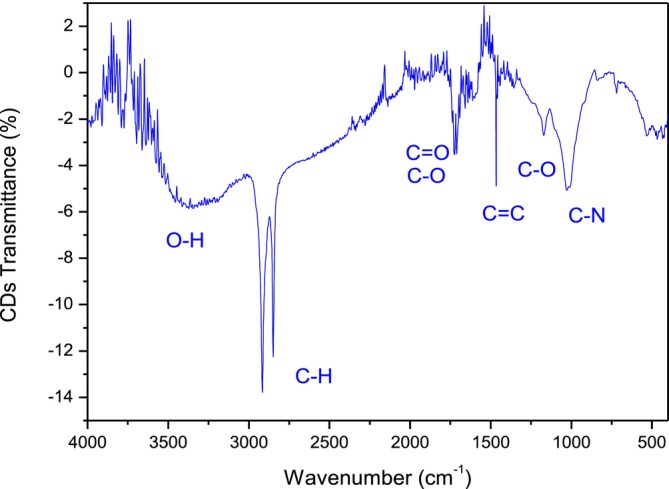
CDs transmittance obtained as FTIR spectra difference in the leaf with and without CDs.

Figure 9 reports the FTIR spectrum obtained by subtracting the leaf spectrum with CDs from that of the virgin leaf, without CDs. Thus, the CDs contain many functional hydroxyl O‐H groups, many C‐H groups, the carbonylic C=O groups, epoxide C‐O‐C groups, double C=C bonds groups, and a significant concentration of cyanide C‐N groups.

The intense bands observed at around 2920 and 2845 cm^−1^, coming from the stretching vibration of C‐H groups, have been measured in intensity as a function of the laser irradiation time, showing a linear increment with this parameter, i.e., indicating an increment of the CDs IR absorbance with their concentration in the dispersion.

**FIGURE 10:**
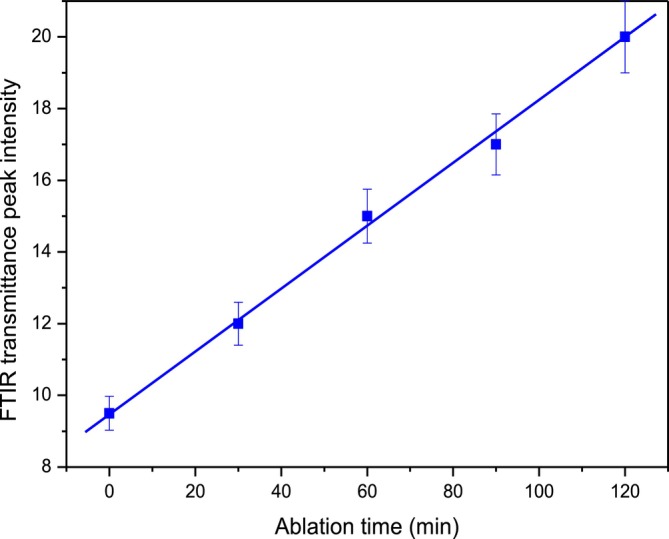
FTIR transmittance intensity peak versus laser irradiation time.

Figure 10 reports the result of the C‐H intensity band measurements at 2920 cm^−1^ versus the laser irradiation time of up to 2 h. The error bars are about 5%. This linearity means that the CDs synthesis enhances nearly linearly with the ablation time.

**FIGURE 11:**
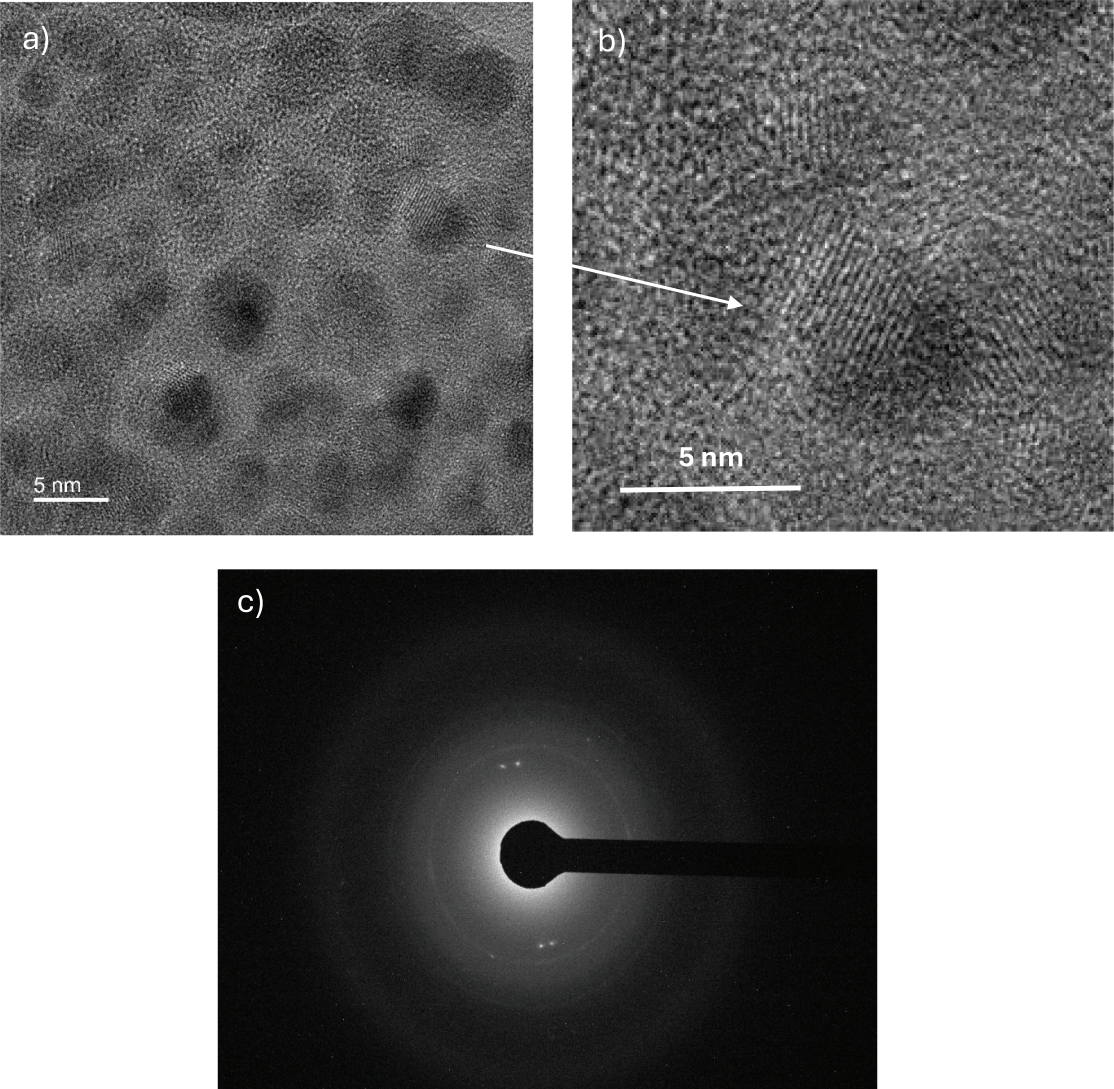
TEM image of the CDs in the dispersion (a), zoom on one crystalline CD (b), and electron diffraction image of the CD (c).

The TEM investigations of the prepared CDs dispersion are summarized in the results reported in the images of Figure 11. The dispersion after 2 h laser irradiation is rich in small nanoparticles, as evident in the first image of Figure 11a, reporting spherical particles with an average distribution size around 2–4 nm. Figure 11b shows a particular part of the previous image showing the ordered atomic structure of carbon atoms. Figure 11c reports an 80 kV electron diffraction image of these CDs, indicating a partial crystalline structure of these nanoparticles. It is assumed that carbon nuclei can be crystalline.

**FIGURE 12:**
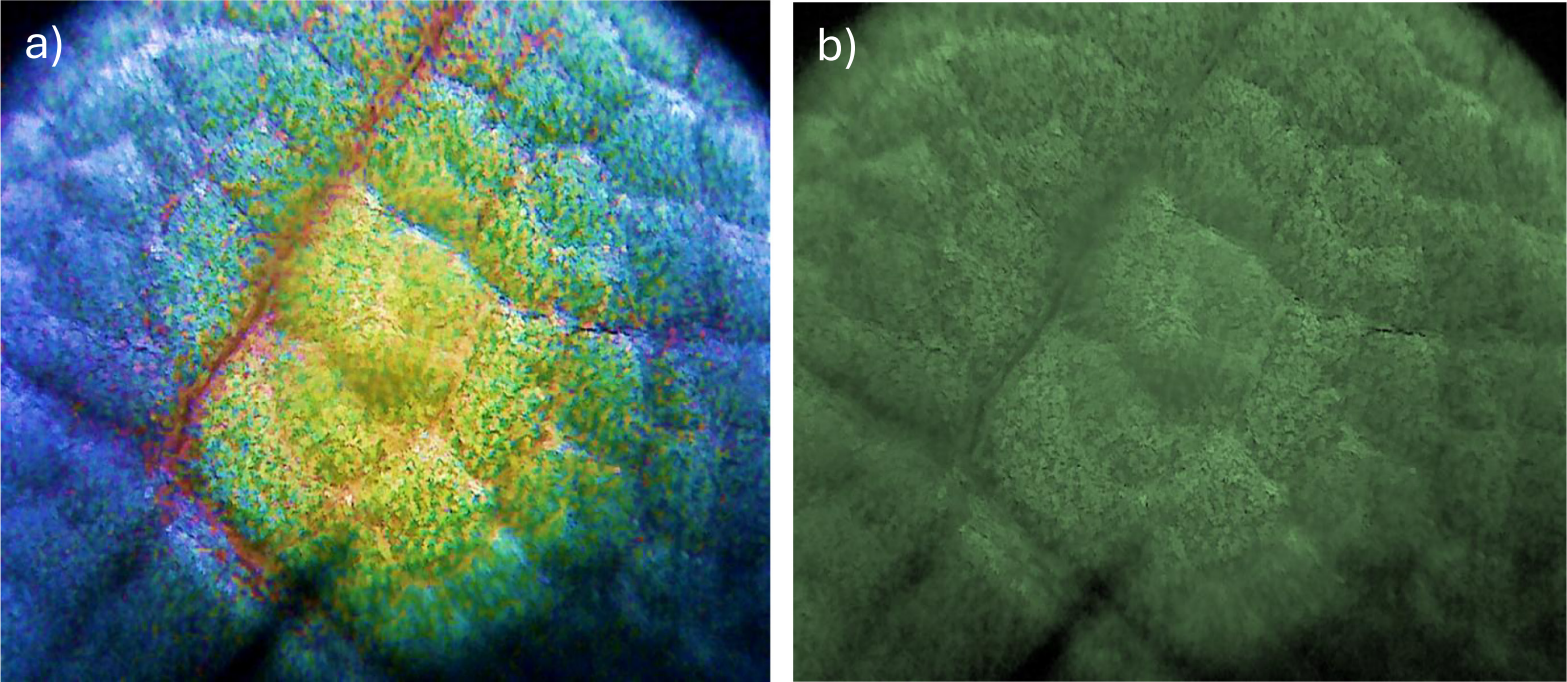
Microscopy of bay leaf with CDs on its surface under UV lamp irradiation (a) and under white lamp irradiation (b).

Further image investigations have been obtained in an optical microscope, illuminating the leaves with a white lamp and a UV lamp without and with CDs nanoparticles. The microscope image reported in Figure 12a was obtained by illuminating with a UV lamp on the front face of the bay leaf containing CDs on the surface, while in Figure 12b the same sample was illuminated with white light. The blue luminescence is well resolved by image comparison.

Previous analyses performed with the laser ablation of vegetable carbon targets (charcoal), and with different lasers, have demonstrated by TEM investigations that the size of the CDs is within 1 and 3 nm, the shape is spheroidal, and the structure is crystalline, as reported in the literature [15, 25]. We hypothesize that, also in this case, the CDs should have a similar shape, size, and structure. Further, SEM and TEM investigations will be presented later to better specify the nature, composition, and structure of the carbon nanoparticles synthesized by the laser ablation with the modalities described here.

The produced CDs suspension by bay leaves kept in the refrigerator at a temperature of around 3°C is very stable even after 3 months.

From the point of view of the exact explanation of the CDs luminescence responsible mechanism, it is actually not simple in our experiment because more investigations must be done. However, there are three possibilities that can be involved in such processes. The first is relative to the CD low size, which, generally, for blue light emission, is of the order of 1–2 nm, and to the carbon crystalline structure, thus comparable in size to the Bohr radius of the exciton of the material. This allows a quantum confinement regime of the exciton inside the nanostructure, which causes variations in the optical properties of the material [26]. The second is relative to the presence of ions in the solution due to both the nature of the bay leaf and to the salts present in the PBS solution that can be bonded to the carbon structure, acting as a dopant. They can be responsible for the electron excitation by UV in higher energetic levels of energy with successive deexcitation and photon emission [27]. The third is due to the possible CDs surface functionalization by simple or complex molecules bonded to the nanostructure surface. Functional oxygen groups, inorganic and organic molecules, aromatic ring molecules, and other molecular structures can be chemically bonded to the carbon surface and be responsible for the electron excitation under UV and consequent deexcitation with photon emission [28]. Such aspects are under study and will be discussed and explored in future work.

Finally, some words about the possible applications of the CDs dispersion. Due to their low cytotoxicity, high solubility in water, high biocompatibility and non‐toxicity, high stability, sensitivity, and selectivity for the emission band depending on the synthesis conditions, high QYs, and fluidity and permeability of the solution, CDs allow their application especially, but not only, in the biological and medical fields [29].

They are excellent candidates for bioimaging, exhibiting broadband absorption and emission covering the far ultraviolet, visible, and near‐infrared bands. They also have high luminescence emission that can be tuned by surface functional species, size, shape, and doping.

In particular, oxygen‐related carboxyl and hydroxyl groups, which confer excellent water solubility without further surface modification, greatly facilitate surface functionalization, with the advantage that CDs may be suitable for drug delivery [30].

The optical and surface CDs properties make them candidates for theranostic applications, such as bioimaging, biosensing, drug delivery, and both in vitro and in vivo studies of cells and tissues. Fluorescence molecular bioimaging plays a key role in tumor detection, allowing observation of pathological and physiological events [4, 31].

CDs can be applied as innovative optical biosensors, even at the single‐cell level, by monitoring the change of fluorescence signals. They can also be used in photodynamic therapy (PDT), photothermal therapy (PTT), and chemotherapy [31].

Their synthesis, as we have exposed in this paper, is very simple, stable, and inexpensive and can be performed with different types of lasers [10, 32], which makes the preparation of laser action a suitable, non‐polluting, and very reproducible technique for obtaining CDs dispersions and their functionalization.

## 4 Conclusions

As from the title, this paper reports on the synthesis of highly fluorescent CDs by laser ablation of bay leaves in biocompatible PBS solutions. The used laser is an ns‐pulsed source at a 1 Hz repetition rate, transporting 100 mJ energy per pulse on about a 1 mm^2^ focused spot. The given laser parameters are optimized in order to enhance the CDs generation in the PBS solution. Another novelty reported by the work concerns the laser ablation not of any leaf but of a laurel leaf, which, as is known, is highly biocompatible and useful in the biomedical field.

The target contains about 440 mg carbon/g and is a vegetable rich in nutrient substances, which free CDs under the ablation process in a liquid. The produced nanoparticles contain hydroxyl O‐H groups, many C‐H groups, carbonylic C=O groups, epoxide C‐O‐C groups, C=C bonds groups, and a significant concentration of cyanide C‐N groups.

The laser leaf ablation is about 1 μg/pulse, while the carbon ablation is about 0.44 μg/pulse due to the high carbohydrate content. Dispersion with a CDs concentration of about 0.39 mg/mL has been obtained.

After 120 min of laser ablation, the CDs dispersed in 8 mL PBS solution, under UV excitation at 365 nm, showed very high PL in the blue visible region, around 472 nm. The first evaluation of the luminescence yield is around 25% or more.

The dispersion liquid is transparent as water under white light, having high transmittance in the visible region, and shows a net blue color under UV irradiation. The luminescence yield is proportional to the laser irradiation time, but for times higher than 2 h, it shows a saturation trend.

The high transmittance, the blue color, and the absorbance peak at 274 nm, as well as the discontinuities presented by the absorbance curve at 300 and 350 nm, indicate that the produced nanoparticles have a size well below 10 nm. CDs with a size of about 1 nm are expected, as in previously cited scientific works of this type.

TEM investigation has evinced spherical CDs shape, with an average size of 2–4 nm, and with a crystalline carbon nucleus.

The presented results are preliminary to the next paper devoted to reporting more information on the TEM images of the CDs and on their surface characterization and composition.

The synthesized CDs have important applications in biology and medicine because they can be used for bioimaging in cell cultures and tissues and can be used for diagnostic and therapeutic aims. Further applications concern their use in optics, radiation detection, sensors, luminescent materials where they can be embedded, and others.

## Conflicts of Interest

The authors declare no conflicts of interest.

## Data Availability

The data that support the findings of this study are available from the corresponding author upon reasonable request.

## References

[1] T. Hirano , K. Kikuchi , Y. Urano , T. Higuchi , and T. Nagano , “Highly Zinc‐Selective Fluorescent Sensor Molecules Suitable for Biological Applications,” Journal of the American Chemical Society 122, no. 49 (2000): 12399–12400.

[2] F. P. G. de Arquer , D. V. Talapin , V. I. Klimov , Y. Arakawa , M. Bayer , and E. H. Sargent , “Semiconductor Quantum Dots: Technological Progress and Future Challenges,” Science 373, no. 6555 (2021): 1–14, 10.1126/science.aaz8541. 34353926

[3] N. Abid , A. M. Khan , S. Shujait , et al., “Synthesis of Nanomaterials Using Various Top‐Down and Bottom‐Up Approaches, Influencing Factors, Advantages, and Disadvantages: A Review,” Advances in Colloid and Interface Science 300 (2022): 102597. 34979471 10.1016/j.cis.2021.102597

[4] G. Nocito , G. Calabrese , S. Forte , et al., “Carbon Dots as Promising Tools for Cancer Diagnosis and Therapy,” Cancers 13, no. 9 (2021): 1991, 10.3390/cancers13091991. 33919096 PMC8122497

[5] L. Torrisi , A. Torrisi , and M. Cutroneo , “Intense Continuous Wave Laser to Synthesize Luminescent Solution of Carbon Dots,” Fullerenes, Nanotubes, and Carbon Nanostructures 32, no. 9 (2024): 866–875.

[6] P. Kaur and G. Verma , “Converting Fruit Waste Into Carbon Dots for Bioimaging Applications,” Materials Today Sustainability 18 (2022): 100137.

[7] D. Ozyurt , M. Al Kobaisi , R. K. Hocking , and B. Fox , “Properties, Synthesis, and Applications of Carbon Dots: A Review,” Carbon Trends 12 (2023): 100276.

[8] X. Wen , P. Yu , Y. R. Toh , X. Ma , and J. Tang , “On the Upconversion Fluorescence in Carbon Nanodots and Graphene Quantumdots,” Chemical Communications 50, no. 2014 (2014): 4703–4706. 24675809 10.1039/c4cc01213e

[9] Y. Liu , Y. Zhao , and Y. Zhang , “One‐Step Green Synthesized Fluorescent Carbon Nanodots From Bamboo Leaves for Copper(II) ion Detection,” Sensors and Actuators B: Chemical 196 (2014): 647–652.

[10] L. Torrisi , L. Silipigni , A. Torrisi , and M. Cutroneo , “Luminescence in Laser‐Generated Functionalized Carbon Dots,” Optics and Laser Technology 177 (2024): 111089.

[11] P. Pavani , K. Kumar , A. Rani , P. Venkatesu , and M. J. Lee , “The Influence of Sodium Phosphate Buffer on the Stability of Various Proteins: Insights Into Protein‐Buffer Interactions,” Journal of Molecular Liquids 331 (2021): 115753.

[12] A. G. Al‐Hashimi and S. A. Mahmood , “The Nutritional Value and Antioxidant Activity of Bay Leaves (*Laurusnobilis* L.),” Basrah Journal of Veterinary Research 15, no. 2 (2016): 246–259.

[13] K. Singletary , “Bay Leaf, Potential Health Benefits,” Nutrition Today 56, no. 4 (2021): 202–208, 10.1097/NT.0000000000000493.

[14] L. Torrisi , A. Torrisi , and M. Cutroneo , “IR Pulsed Laser Ablation of Carbon Materials in High Vacuum,” Applied Sciences 14 (2024): 11744.

[15] L. Torrisi , M. Cutroneo , L. Silipigni , et al., “Luminescent Carbon Dots Structure by Charcoal Laser Ablation in Biocompatible Liquid,” Fullerenes, Nanotubes, and Carbon Nanostructures (2025): 1–11, 10.1080/1536383X.2025.2461504.

[16] S. Mediavilla , A. Garcia‐Ciudad , B. Garcia‐Criado , and A. Escudero , “Testing the Correlations Between Leaf Life Span and Leaf Structural Reinforcement in 13 Species of European Mediterranean Woody Plants,” Functional Ecology 22 (2008): 787–793.

[17] “Density of Bay Leaves, Actual Website 2025: Density of Bay Leaves, UPC: 041313028138 In 285 Units And Reference”.

[18] “Medicago, Phosphate Buffered Saline (PBS),” actual website 2025, https://www.medicago.se/sites/default/files/pdf/productsheets/PBS_Buffer_v._01.pdf.

[19] Sigma‐Aldrich , “PBS Tablets, Actual Website 2025,” https://www.sigmaaldrich.com/IT/it/product/sial/08057.

[20] Q. Rong , J. Liu , Y. Cai , et al., “Leaf Carbon, Nitrogen and Phosphorus Stoichiometry of *Tamarix chinensis* Lour. in the Laizhou Bay Coastal Wetland,” Ecological Engineering 76 (2015): 57–65.

[21] M. Liu , “Optical Properties of Carbon Dots: A Review,” Nanoarchitectonics 1, no. 1 (2020): 2, https://wiserpub.com/uploads/1/20220428/eaa6e937f2580c594f16c9ab3809fc9e.pdf.

[22] O. Dimitriev , D. Kysil , A. Zaderko , et al., “Photoluminescence Quantum Yield of Carbon Dots: Emission due to Multiple Centers Versus Excitonic Emission,” Nanoscale Advances 6 (2024): 2185–2197. 38633041 10.1039/d4na00033a PMC11019485

[23] N. Hoda , L. B. Akpolat , F. M. Sivri , and D. Kurtuluş , “Biosynthesis of Bimetallic Ag‐Au (Core‐Shell) Nanoparticles Using Aqueous Extract of Bay Leaves (*Laurus nobilis* L.),” JOTCSA 8, no. 4 (2021): 1035–1044.

[24] “Infrared Spectroscopy Correlation Table,” actual website 2025, https://en.wikipedia.org/wiki/Infrared_spectroscopy_correlation_table.

[25] A. Kurdekar , L. A. A. Chunduri , E. P. Bulagonda , M. K. Haleyurgirisetty , V. Kamisetti , and I. K. Hewlett , “Comparative Performance Evaluation of Carbon Dot‐Based Paper Immunoassay on Whatman Filter Paper and Nitrocellulose Paper in the Detection of HIV Infection,” Microfluidics and Nanofluidics 20, no. 7 (2016): 99, 10.1007/s10404-016-1763-9.

[26] D. Bera , L. Qian , and T. K. Tseng , “Quantum Dots and Their Multimodal Applications: A Review,” Materials 3, no. 4 (2010): 2260–2345.

[27] R. Bandi , B. Reddy , R. Dadigala , R. Eslavath , S. Singh , and V. Guttena , “Facile and Green Synthesis of Fluorescent Carbon Dots From Onion Waste and Their Potential Applications as Sensor and Multicolour Imaging Agents,” Advances 6, no. 34 (2016): 28633–28639, 10.1039/C6RA01669C.

[28] B. B. Chen , M. L. Liu , C. M. Lia , and C. Z. Huang , “Fluorescent Carbon Dots Functionalization,” Advances in Colloid and Interface Science 270 (2019): 165–190. 31265929 10.1016/j.cis.2019.06.008

[29] V. B. Kumar , S. K. Mirsky , N. T. Shaked , and E. Gazit , “High Quantum Yield Amino Acid Carbon Quantum Dots With Unparalleled Refractive Index,” ACS Nano 18, no. 3 (2024): 2421–2433. 38190624 10.1021/acsnano.3c10792 PMC10811667

[30] W. Su , H. Wu , H. Xu , et al., “Carbon Dots: A Booming Material for Biomedical Applications,” Materials Chemistry Frontiers 4 (2020): 821–836.

[31] L. Torrisi , A. Torrisi , D. Cosio , and M. Cutroneo , “Optical Properties of Carbon Dots Generated in Liquid by Pulsed IR Laser at 1064 nm,” Fullerenes, Nanotubes, and Carbon Nanostructures 33, no. 5 (2024): 495–508, 10.1080/1536383X.2024.2424346.

[32] L. Torrisi , A. Torrisi , and M. Cutroneo , “Carbon Dots Synthetization by Intense CW Laser at 450 nm,” Nanoparticle 5, no. 1 (2014): 10014, 10.35702/nano.10014.

